# No Country for Young Refugees: Barriers and Opportunities for Inclusive Refugee Education Practices

**DOI:** 10.1177/10591478241243382

**Published:** 2024-04-16

**Authors:** Sebnem Manolya Demir, Feyza G Sahinyazan, Bahar Y Kara, Elfe Buluc

**Affiliations:** 1 Marshall School of Business, 5116 University of Southern California,Los Angeles, CA, USA; 2 Beedie School of Business, 1763 Simon Fraser University, Vancouver,BC, Canada; 3 Department of Industrial Engineering, 52948 Bilkent University, Ankara, Türkiye; 4 United Nations High Commissioner for Refugees (UNHCR) Türkiye, Ankara, Türkiye

**Keywords:** Refugee crisis, access to education, inclusion, social justice, refugee integration, maximal covering problem

## Abstract

The recent refugee crises in Ukraine (2022) and Syria (2011) have created millions of refugees, 40% of whom are children. The education systems of countries hosting refugees struggle to integrate such large populations. In addition, language barriers and the stigma associated with refugees hamper inclusive and equitable education opportunities for these children. There is thus a risk of “lost generations” distanced from education, who may eventually depend on social security systems and monetary aid in the long term. This study considers the following research question: How can a host country improve the inclusion of refugee children in the education system without overburdening its infrastructure? First, we document the availability and accessibility challenges and opportunities that refugee children face during the Syrian refugee crisis. We then develop an inclusive planning strategy aligned with existing capacity and resources and formulate two adaptations of the maximum covering problem (MCP): cooperative capacitated MCP with heterogeneity constraints (CCMCP-HC) to improve the current schooling access in Türkiye and Modular CCMCP-HC to guide early planning in the case of a future crisis. Our computational analyses illustrate that the proposed approach yields higher schooling rates and capacity utilization than existing approaches. Our results emphasize the importance of having a planning strategy in the initial phases of a crisis that considers future integration possibilities. This study analyzes Türkiye’s experience and lessons learned to provide a road map for other ongoing and future refugee crises.

## 1. Introduction

There are currently 110 million forcibly displaced individuals globally (UNHCR, 2023), and the UNHCR estimates that another 100 million will join them by 2050 due to conflicts and climate crises (UNHCR, 2016). The Syrian Civil War started in 2011, and with more than half a million casualties and 6.6 million refugees, it has been named the largest humanitarian crisis of our time (UNHCR, 2021c). Türkiye (formerly referred to as Turkey) hosts the world’s largest refugee population, with 98.6% living outside of camps across the country in primarily urban settings. Among the 3.6 million Syrian refugees living in Türkiye, 1.6 million are children, many of whom have suffered intense emotional trauma. In early 2022, Russia invaded Ukraine, displacing 6 million people, half of whom were children, leading them to seek refuge in neighboring countries, including Poland, Hungary, and Romania (UNHCR, 2022).

Refugees are often considered a burden to their host communities. However, hosting refugees can bring benefits, especially when they are well-educated and integrated into their new home (Jacobs, 2017; Salehyan, 2018); refugees outperform other migrants in labor markets, paid taxes, and language proficiency (Cortes, 2004; Evans and Fitzgerald, 2017) and recent evidence shows benefits to youth in the host country as a result of increased educational opportunities and competition in the labor market (Tumen, 2021). Yet, most refugees face discrimination and exclusion when they arrive in a new country, including in education (Kroon and Paauwe, 2014; Shamieh et al., 2022).

Children make up nearly 40% of the refugee population (UNHCR, 2021b) and are five times more likely to be out of school (UNICEF, 2017). Disruptions in refugee children’s education tend to become permanent, as the crisis that displaced them may not stabilize for decades. Being distanced from employment opportunities, conflict-impacted parents often encourage their children to take up low-paying employment, trapping them in an indefinite poverty cycle (UNICEF, 2014). In addition, the evidence on the most effective ways to ensure inclusive educational opportunities is mixed and limited (de Hoop et al., 2019). The question of how to effectively integrate refugee children into the host country’s educational systems in the short and long term remains unanswered (UNHCR, 2015). It is critical to ensure these future adults receive the emotional support, education, and training they need to succeed later in life (UNHCR, 2021a). However, providing such opportunities equitably can result in significant capacity shortages for the host country. In this study, we explore these challenges in answering the following research question: *How can a host country improve the inclusion of refugee children in its education system without overburdening its capacity?* We begin by considering the Syrian refugee crisis to examine the barriers and opportunities encountered by policymakers. We then propose mathematical models that aims to increase educational availability and accessibility for refugee children while respecting local regulations.

The contributions of this study are threefold. First, to the best of our knowledge, it is the first operations management study to focus on the educational inclusion of refugee children. While the United Nations Sustainable Development Goals (SDGs) ignite a growing interest among the OR/MS community, SDG 4 (Quality Education) is often overlooked (Besiou et al., 2021). Meeting these goals requires considering the well-being of millions of refugees (Corbett et al., 2022).

Second, rather than solely focusing on the objectives of the Turkish Ministry of National Education (MoNE) and humanitarian organizations, we propose solutions based on the documented concerns and challenges faced by beneficiaries. The dominant framing of research in refugee settings tends to ignore the voice and agency of refugees (Vigneswaran and Quirk, 2013). Our results show that reevaluating existing guidelines and policies developed before the arrival of refugees is crucial for the sustainability of solutions.

Finally, we introduce two new mathematical formulations to the logistics domain: cooperative capacitated maximal covering problem with heterogeneity constraints (CCMCP-HC) and modular CCMCP-HC. We develop the CCMCP-HC to improve the educational situation in Türkiye, where the location of most schooling options and capacities were haphazardly decided; neither the Turkish government nor refugees have planned for long-term integration. The modular CCMCP-HC is developed to support host countries in taking proactive decisions and allocating capacity in modules as future crises unfold. Maximal covering problems are widely used in public-sector network design; however, the commonly used variants have very restrictive assumptions that prevent practical and realistic solutions. Our results show that, compared to existing approaches, our proposed models would significantly enhance capacity utilization and schooling availability.

The remainder of this article is organized as follows: in Section 2, we review the literature on refugee operations, refugee schooling, and capacitated maximum covering models; in Section 3, we outline the problem context, historical efforts in the education of refugee children, and detail existing challenges. In Section 4, we introduce the proposed mathematical models and share the computational results for three Turkish provinces. In Section 5, we conclude and suggest avenues for future research.

## 2. Literature Review

### 2.1. Refugee Crises Response Operations

The operations management literature on refugee contexts is rather limited (Oloruntoba and Banomyong, 2018). While only 20% of refugees live in refugee camps, most studies in this domain focus on camp operations. Jahre et al. (2018) is one of the first to provide an analytical framework for camp design, using case studies of UNHCR camps constructed for Syrian refugees in Greece and Türkiye. Karsu et al. (2019) introduce an algorithm for refugee camp layout optimization, and Bravo et al. (2019) develop a path-planning optimization model for drones to video map potential camp locations.

Once a camp design is established, the next step is to supply aid effectively (Balcik et al., 2016). McCoy and ML Brandeau (2011) propose a relief-item shipment policy for UNHCR and Jahre et al. (2016) develop a network optimization model that integrates UNHCR’s emergency and long-term operational supply chains to reduce logistics costs. Ergun et al. (2014) explore how information technologies can facilitate coordination among humanitarian organizations in a refugee camp through a game theory model.

Two notable studies explore the dynamics between refugee camps and adjacent populations. Azizi et al. (2021) develop an inventory management model that balances the aid delivered to a refugee camp and the surrounding refugee population. Kotsi et al. (2022) analyze the effect of cash distributions provided to refugees on the prices of commodities and provide a pricing mechanism that can protect the host community from price inflation. Few studies focus on the refugee resettlement problem, that is, assignment of refugees to the most suitable neighborhoods to increase their chances of adaptation to their new country (Bansak et al., 2018; Ahani et al., 2021). The urban integration of the refugees without overburdening the existing service systems of the host countries is yet to be studied. For a more detailed review of the operations management literature in response to refugee crises, see Seifert et al. (2018).

### 2.2. Refugee Children’s Educational Practices

Successful strategies for the classroom-level integration of refugee children include mainstreaming (i.e., a few refugee children included in each class) and small group instruction (Hamilton and Moore, 2004; Arar, 2021). However, these options are only feasible if the host country has ample capacity and resources (e.g., New Zealand and Canada). As a highly successful but exceptional case, Finland designed individual curricula in student–family–teacher collaborations (Dervin et al., 2016); this was possible thanks to Finland’s long-term commitment to educational excellence and the low number of refugees in the country.

In the Syrian refugee crisis, even with significant resources and long-term immigration expertise, many European countries struggled with the massive influx of refugees, relying on ad-hoc measures (Koehler and Schneider, 2019). In many cases, refugee students’ success is hampered by aged-based central exams, with students having to adopt new syllabi in a foreign language while dealing with loss and trauma. In particular, McIntyre and Hall (2018) conclude that educational best practices cannot be consistently implemented at the desired levels, especially if this is done employing high-stakes exams. Relaxing age or system-based constraints designed with the host country’s students in mind can yield beneficial results. Italy allowed refugees to obtain official school certificates even if they passed the age limit (Grigt, 2017). Similarly, Korntheuer (2022) finds that refugees in Canada benefit from relaxed age restrictions, whereas stricter programs prevail in Germany.

Crul et al. (2019) compare the integration of Syrian refugee children in five countries: Germany, Greece, Lebanon, Sweden, and Türkiye. Germany and Sweden already had ongoing second-language pedagogical practices for occasional immigrants. Furthermore, the refugee parents in these countries were found to be more cooperative, as those countries are seen as final destinations. Traveling to Europe requires ample financial resources and poorer and less educated families were stuck in Greece, Lebanon, and Türkiye; these countries, with fewer resources and less immigrant expertise, had to oversee significantly more refugees than Sweden or Germany. In all three countries, the public and refugees perceived the situation as temporary, and the response was thus substantially delayed.

After a refugee child is allocated to a learning option, it is essential to ensure their continued attendance. de Hoop et al. (2019) explore the impact of a pilot cash aid program, “No Lost Generation,” implemented for Syrian refugees in Lebanon by UNICEF. The authors conclude that the limited success of the program could be linked to the limited schooling capacity available for new enrollments. This is consistent with findings in the literature; systematic reviews and meta-analyses of the cash programs demonstrate the success of such programs is somewhat limited (6% improvement in school enrollment and 3% in attendance (Doocy and Tappis, 2017)). Accordingly, in their meta-analysis, Burde et al. (2015) suggest that strengthening schooling capacity and providing continued access to the education systems can be the only sustainable option for properly integrating refugee children.

Although several descriptive studies document the in-class experience of refugees, few provide guidelines for effective capacity expansion and inclusion in district or country-level planning. Integrating refugee children should not mean holding them to standards that are not designed for vulnerable populations. Furthermore, the host country’s language programs should be supported by teaching staff that have adequate expertise in educating traumatized children. Finally, it is time for the hardships of the host countries and their overburdened systems to be recognized and decision support systems developed to ease rapid and effective transition.

In our study, we tailored our recommendations, keeping the documented lessons learned in mind and our proposed systems can enable the following: (i) More rapid inclusion of refugees to increase their sense of belonging to the “transit countries.” Rather than trying to follow the native curricula of refugees, our models propose that students enroll in the host country’s schools as soon as possible. (ii) Support students through teachers with adequate pedagogical skills that can handle children who experience trauma and loss. Depending on the scale of the problem, this could be a very scarce resource. In our study, we focus on solutions to increase scarce capacity utilization. (iii) Reduce segregation. In many cases, it has been shown that relaxing constraints initially designed for the host country’s youth might be necessary. In our context, we recommend relaxing address-based requirements for refugee children and demonstrate its effects through computational analysis of capacity availability and utilization.

### 2.3. Capacitated Maximum Covering Models

The MCP is first developed to find the optimal locations for public facilities. The initial model variant was developed to find the maximum population that can be covered with a fixed number of facilities. This model is defined with binary assignment constraints, representing a single service level, within which a given population is either fully covered by a facility or not covered at all, commonly referred to as *all-or-nothing* coverage. The same binary constraints also presume that every demand point can only be covered by a single (usually nearest) facility, namely *individual* coverage. Furthermore, the considered facilities are *uncapacitated*, meaning the facility can cover any demand point within the coverage radius. Additionally, all the facilities are assumed to be identical. Finally, all agents traveling to the facility are assumed to use the same mode of transportation.

As these assumptions are too restrictive for practical applications, numerous extensions of the maximal covering model are introduced. The generalized maximal covering model is defined to explore the cases with *multilevel service*, where different service types are possible at different service levels (Church and Roberts, 1983; Berman and Krass, 2002; Eiselt and Marianov, 2009) instead of all-or-nothing coverage. Similarly, new extensions are studied for cases where multiple facilities can cover the same demand point *cooperatively*. Berman et al. (2010) note that dichotomous all-or-nothing models are not good representations of real life. Berman et al. (2019) further relax this constraint, imposing complete demand coverage. Álvarez-Miranda and Sinnl (2019) develop an exact algorithm for this problem.

**Table 1.** Contribution of cooperative CMCP-HC.

	A versus ML	I versus C	Capacitated facilities	Heterogenous facilities	Heterogenous mode of transportation
Álvarez-Miranda and Sinnl (2019)	ML	C			
Delmelle et al. (2014)	ML	C	+		
Berman and Krass (2002)	ML	I			
Berman et al. (2010)	ML	C			
Berman et al. (2019)	ML	C			
Chang and Liao (2015)	ML	I	+		+
Church and Roberts (1983)	ML	I			
Eiselt and Marianov (2009)	ML	I			
Heyns and Vuuren (2018)	A	C		+	
Karatas (2017)	ML	C	+		
Pirkul and Schilling (1991)	ML	I	+		
Wang et al. (2016)	ML	I	+	+	
Yin and Mu (2012)	ML	I	+	+	
Our contribution	ML	C	+	+	+

Note. CMCP-HC = capacitated maximum covering problem with heterogeneity constraints; A = all-or-nothing coverage; ML = multilevel service; I = individual coverage; C = cooperative coverage.

Public facilities such as hospitals and schools require *capacitated* formulations, as their service capacity can be a critically binding factor (Pirkul and Schilling, 1991). More recent examples for emergency vehicle networks allow capacity to be determined in a modular format (Yin and Mu, 2012). Delmelle et al. (2014) use a similar approach for expanding the existing schooling capacity to meet increasing demand, and Karatas (2017) combines cooperative and multilevel service through capacitated facilities and tests the algorithm performance on synthetic data sets.

MCP traditionally focuses on a single facility type, and the literature on *heterogenous facility* location with maximal covering is limited. Wang et al. (2016) determine the fire stations opened within a limited budget, with each station having different hazard response capabilities. Heyns and Vuuren (2018) consider a multitype multi-zone coverage problem, where different facilities can be located in different zones. While several studies allow heterogeneous modes of student transportation (i.e., walking, biking, and school buses) (Kim et al., 2012; Easton and Ferrari, 2015; Sales et al., 2018; Bertsimas et al., 2019), these studies consider routing and scheduling decisions in isolation. Chang and Liao (2015) is one notable example in which heterogeneous transportation modes are introduced within the MCP; the authors design a network of emergency shelter locations and estimate the coverage depending on the evacuees’ potential walking, driving, and biking behavior. To the best of our knowledge, no formulation in the literature combines all these realistic extensions simultaneously. We aim to fill this gap by developing a formulation that allows multilevel service and cooperative coverage through capacitated but heterogeneous facilities with heterogeneous transportation modes. The existing literature and our contribution are compared in Table 1.

## 3. Barriers and Ongoing Efforts to Syrian Refugee Children’s Education

In this section, we first summarize the documented challenges faced by various stakeholders (i.e., children, parents, teachers, humanitarian organizations, and the government) in responding to the Syrian refugee crisis. We then explain diversity, equity, and inclusion (DEI) efforts in refugee schooling and set out the key differences between our approach and existing practice.

### 3.1. Ongoing Efforts

During the early phases of the Syrian crisis response (2011–2014), ad-hoc education programs were administered in Temporary Education Centers (TECs) located in refugee-dense districts of Türkiye. Instruction in these centers is given in Arabic by Syrian educators (Brugha et al., 2021). The main goal was to follow the Syrian curriculum so that on their return to Syria, these children would not have fallen behind in their education.

While Syrian refugees had the right to enroll in Turkish state schools since 2011, in the absence of Turkish language training and bridging programs to close the curricula gap, refugees and the Turkish government were reluctant to large-scale uptake. This hesitancy resulted in limited financial and social investment in capacity expansion (Landau and Studies, 2016).

Around 2016, it became evident that the situation was not temporary. The Turkish MoNE declared its intention to fully integrate refugees into the formal national education system (UNESCO, 2018) by closing the TECs and extrapolating the existing address-based school assignment policies applicable to Turkish children to refugees. MoNE has since trained 5,200 Turkish language teachers to teach Turkish to Syrian children, and 1,800 school principals have attended seminars on the inclusion of Syrian students (European Commission, 2017). Finally, conditional and unconditional cash transfer programs have been implemented to increase school attendance.

However, these initial nudges can only be leveraged for long-term success by ensuring sufficient schooling capacity and more inclusive schooling systems (Brugha et al., 2021; Aygün et al., 2021). The “double-shift” programs where half of the children are taught in the morning, and the rest are taught during the afternoon—already common for Turkish children—were made available to Syrian children. Teachers overseeing Syrian students are expected to make pedagogical provisions for working with traumatized children; however, few MoNE teachers have such qualifications. Accordingly, a pilot program—Trauma-Informed Teaching Initiatives—was introduced to train teachers in supporting Syrian children who had experienced war and loss (Theirworld, 2021).

### 3.2. Barriers to Refugee Student Integration

Since the earliest phases of the crisis, UNICEF (2014) warned against the risk of “lost generations” due to an increase in school drop-out rates, reinforcing the poverty trap for future generations. Despite all efforts, there are 400,000 out-of-school refugee children in Türkiye (UNICEF, 2018). The situation is even worse for high-school-aged refugees; 45.1% are involved in child labor, and only about 3% are enrolled in school (Dayioglu et al., 2021).

Teachers working with Syrian children report that a lack of appropriate pedagogical and language formation negatively impacts their success and motivation (Seker and Sirkeci, 2015). There have also been reports of bullying and discrimination against refugee children due to teachers’ inexperience in managing multicultural classrooms. These incidents have reduced the willingness of Syrian families to send their children to state schools (Erdogan, 2014). However, the lack of schooling capacity continues to be the main barrier, especially if TECs are shut down (Coskun and Emin, 2016). Shamieh et al. (2022) interviewed Syrian parents to identify the obstacles hindering educational access, reporting the three most cited concerns as overcrowded classrooms, exposure to bullying, and lack of transportation.

The plan to close the TECs was controversial. While double-shifted state schools can increase enrollment rates by reducing the concerns over bullying and discrimination, the spatial inconvenience of these state schools remained intact compared to the TECs’ proximity. The TECs were initially located in the provinces’ refugee-dense areas and were responsible for numerous students. Since the distance to school is a critical constraint for Syrian refugees’ schooling, shutting down TECs is expected to further reduce attendance (Coskun and Emin, 2016; Erdogan, 2014). On the other hand, TECs could not grant official diplomas, as TEC curricula were not designed or approved by the MoNE.

In 2017, the World Bank announced a three-year support project for Türkiye’s education infrastructure with a total budget of 157 million dollars (World Bank, 2017). The initial budget included a 14% allocation to infrastructure at the secondary school level ( ∼ 400 classrooms). However, by the end of the original closing date (June 2019), only 6.4% of the budget has been deployed (World Bank, 2021). Given the disruptions in plans to expand state school capacity, the closure of TECs will further decrease refugee children’s access to school. Importantly, the process for determining the locations and capacities of these schools funded by the World Bank is not described or analyzed in either report. Furthermore, no policy changes to increase existing capacity other than building schools are considered.

### 3.3. DEI in Refugee Schooling

There is limited DEI know-how within the Turkish MoNE, which currently makes entirely “address-based” decisions on school assignments and transportation requirements. That is, all children residing in a schooling district are assigned to the same school through the “e-school” system (MoNE, 2014). The process is intended to assign everyone to their nearest option and not consider the ethnicity, race, gender, or socio-economic state of the families. A recent DEI application —inclusive classrooms— was introduced for children with special education needs (visual, hearing, physical disabilities, attention-deficit/hyperactivity disorder, or on the autism spectrum). However, at most, two children with special needs can be assigned to the same classroom, and the equitable distribution of these options among all classrooms or schooling districts is not considered (MoNE, 2018).

DEI efforts in school assignments are built around one central theme: reducing segregation by redesigning schooling districts in which, compared to national averages, students from certain backgrounds are disproportionately represented. There is a vast OR/MS literature on the desegregation of schools in the U.S. after the country’s Supreme Court outlawed school segregation (Heckman and Taylor, 1969; Belford and Ratliff, 1972; Liggett, 1973; Ferland and Guénette, 1990; Liao et al., 2020; Wei et al., 2022). These studies focus on address-based systems in school districts, with a common objective to achieve zoning that ensures demographic homogeneity. In almost all cases, these efforts produce gerrymandered school zones: noncompact districts with complex geometries that can increase commute times (Sydänlammi, 2019; Wei et al., 2022). Furthermore, empirical evidence shows that gerrymandering causes further segregation in regions that are rapidly diversifying (Richards, 2014).

Another potential approach, overlooked in the literature, is working with continuous decision variables to split neighborhoods (cooperative coverage) into different schools. Caro et al. (2004) explain why splitting solutions are not popular; these solutions increase the number of decisions to be made (e.g., which students will be assigned to schools further away). While these concerns are certainly valid in the context of addressing racial segregation in the U.S., where neighborhood demographics are more stable and school budgets are determined at the district level, these approaches may not be extrapolated to refugee contexts. For example, public school budgets in Türkiye are determined centrally and based on school capacity.

Studies in Europe offer relevant insights due to the large populations of refugees. Many European countries have a policy of free school choice: students can apply to any school within their municipal borders and will be accepted if there is space. However, the schools must first secure capacity for the children within their zones. At first glance, this system might yield more equitable and diverse schools since immigrants are not forced to attend schools in immigrant-dense zones. However, all evidence points to the contrary: free school choices not only fail to remedy segregation but worsen it (see Yang Hansen and Gustafsson (2016) for Sweden and Kosunen (2014) for Finland). Local populations tend to migrate away from refugee-dense zones within the city (Sydänlammi, 2019). An alternative would be to introduce lotteries to assign excess capacity so that families can still apply to their preferred school; however, even a lottery system would not overcome selective in-city migration and, in addition, increase students’ total commute time (Liao et al., 2020). For a comprehensive analysis of different school assignment policies, see Smilowitz and Keppler (2020).

### 3.4. Proposed Approach

Various schooling systems have been considered as options for overcoming segregation, but existing studies are inconclusive. In our research, instead of expanding the address-based assignment to refugee children, we recommend that Türkiye assign refugee children across schools with sufficient capacity through cooperative coverage. This would prevent the following: (i) underutilization of capacity for Syrian children (Section 4.2.1) and (ii) further segregation, especially in refugee-dense neighborhoods, due to selective in-city migration dynamics. Turkish parents are reportedly changing their addresses to ensure their children attend their preferred schools (Ikamet, 2021; Buyruk, 2020), enabling a de facto free-choice system similar to that operating in European cities.

This study addresses these barriers through a planning strategy to help decision-makers design more inclusive education opportunities without burdening existing education capacity. The suggested approach enables the optimized use of available resources and funds, such as those provided by the World Bank. Accordingly, we propose a hybrid system combining the benefits of TECs and state schools for increased schooling availability. We suggest incorporating TECs into a host country’s formal schooling system. For this, we develop mathematical models that determine the optimal set of TECs to be integrated into the host country’s system (these we refer to as “transformed TECs” or tTECs), the optimal set of state schools for refugee children to enroll in, their capacities, and children’s assignments to these options. We also recommend relaxing address-based assignments to increase capacity utilization and reduce segregation. Finally, we consider school transportation for increased accessibility; for a more detailed discussion on school transportation policies in Türkiye, see Online Appendix A.

## 4. Mathematical Models and Results

In this section, we present two capacitated maximal covering models for increased refugee schooling rates and capacity utilization, and then summarize our computational analyses.

### 4.1. Model Descriptions

Our models decide on the optimal education capacity (i.e., budget and teaching staff), location, and allocation in the presence of TECs and state schools. Two types of educational facilities introduce heterogeneous facilities to the problem. The models also consider heterogeneous modes of transportation; there are two transportation alternatives for refugee children: walking and using the school bus. In alignment with government regulations, our models determine the modes of transportation between each refugee district and the school it is assigned to. Differences in the allowable distances for different transportation modes require multilevel service.

First, we present the CCMCP-HC, which combines multilevel service and cooperative coverage approaches. In contrast to classical maximal covering models and the approach of address-based school assignment practices, we allow cooperative coverage. In terms of cooperative coverage, children in each district can be partially assigned to different schools. For this model, we use integer decision variables representing the number of children assigned to a particular school rather than the classical binary decision variables of MCPs. This model allows us to examine potential improvements given Türkiye’s existing education infrastructure. We then include capacity-allocation decisions through modular capacity assignments (modular CCMCP-HC) to increase schooling access rates and the utilization of critical resources, such as bilingual teachers with a proper background in trauma management. The latter model can serve as a planning tool for the early stages of a crisis (e.g., the current situation in Ukraine) for decision-makers who want to adopt a proactive educational system strategy for refugees rather than an ex-post approach. For brevity, we provide the CCMCP-HC and modular CCMCP-HC formulations in Online Appendixes B.1 and B.2, respectively.

### 4.2. Computational Analyses

In our computational analyses, we employ data from three Turkish provinces—Kilis, Gaziantep, and Sanliurfa—adjacent to the Syrian border. All formulations are solved using the IBM ILOG CPLEX Studio 12.8, and the computational times for all instances are less than a minute, thus, the computational performances are not reported separately. A detailed description of the data sets and parameter estimation procedures are presented in Online Appendix C.

#### 4.2.1. Shortcoming of Address-Based Assignment Policy

The Turkish MoNE uses address-based school assignments, with all children resident in a school district assigned to the nearest school through the “e-school” system (MoNE, 2014). This system has multiple advantages in the *business-as-usual* case, including reducing transportation costs and preventing before- and after-school traffic. However, the system was not designed in anticipation of a surge in schooling demand.

**Figure 1.**
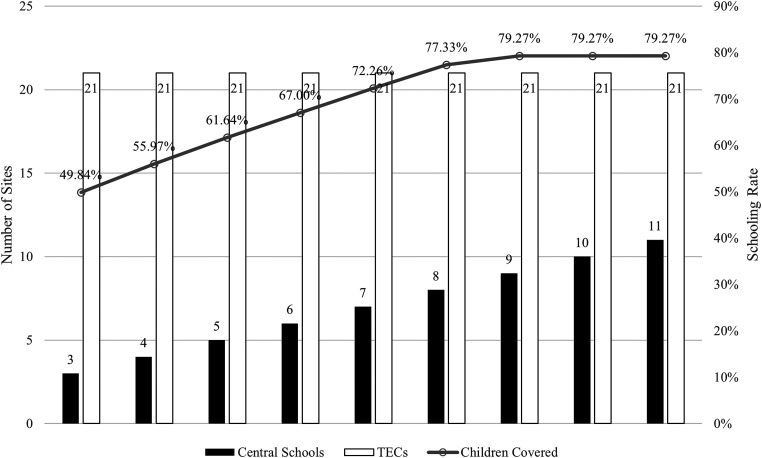
Number of Kilis TECs to transform to achieve maximum coverage under address-based policy.

In Figure 1, the left-hand side vertical axis represents the total number of schools opened for a given scenario, that is, the number of tTECs that should be utilized (white bars) for the given number of central schools (black bars) in Kilis province. The right-hand side vertical axis demonstrates the maximum coverage achievable under the address-based policy and the composition of central schools and tTECs.

Kilis currently has 15 state schools, three selected as central schools to host refugee children and 21 TECs. The current capacity yields a 49.84% schooling rate with a capacity utilization of 73%, even if these allocations are made optimally. Our results show that utilizing TECs while supporting central schools with adequate resources improves overall rates of schooling over the status quo. However, these results also underline the shortcomings of the address-based policy. Under this restriction, schooling rates plateau after nine central schools are added to the system, and a further increase in capacity would result in underutilization. Even though more than half of the children are not schooled, unused capacity exists due to this policy. Our results also show that additional capacity (beyond nine central schools and 19 tTECs) does not increase school enrollment. Thus, our first recommendation is as follows: in a crisis where resources are scarce, it is crucial to rethink existing regulations to ensure higher capacity utilization and schooling coverage.

#### 4.2.2. Performance of Proposed Models

We formulate the CCMCP-HC with integer assignment decision variables representing the number of children rather than a binary assignment of address-based policy. In this model, children from the same district could be covered by different schooling options through coordination. In Table 2, we compare the results of these formulations concerning maximum coverage and utilization. For each instance, the CCMCP-HC yields higher schooling rates than the current policy—due to the higher capacity utilization achieved by cooperative coverage. This policy change would increase schooling rates from the status quo coverage of 49.84% to 65%, with a 95% capacity utilization. More importantly, a 100% coverage rate is possible with nine central schools and 19 tTECs, compared to schooling rates converging to 79.27% in address-based assignments.

**Table 2.** Comparison of coverage and utilization rates obtained by address-based, CCMCP-HC, and Modular CCMCP-HC assignments.

		Address-based		CCMCP-HC		Modular CCMCP-HC	
Central schools	tTECs	Coverage (%)	Utilization (%)	Coverage (%)	Utilization (%)	Coverage (%)	Utilization (%)
3	21	49.84	73.17	65.35	95.95	68.11	100.00
4	21	55.97	75.26	71.61	96.29	74.37	100.00
5	21	61.64	76.45	77.88	96.58	80.63	100.00
6	21	67.00	77.10	84.14	96.83	86.90	100.00
7	21	72.26	77.56	90.40	97.04	93.16	100.00
8	21	77.33	77.78	96.67	97.23	99.42	100.00
9	19	79.27	78.50	100.00	99.02	100.00	99.02
10	16	79.27	79.11	100.00	99.80	100.00	99.80
11	14	79.27	77.89	100.00	98.26	100.00	98.26

Note. CCMCP-HC = cooperative capacitated maximum covering problem with heterogeneity constraints; tTECs = transformed Temporary Education Centers.

The underutilization of scarce resources is almost entirely avoided when we abandon address-based coverage. Nevertheless, the CCMCP-HC does not achieve 100% capacity utilization since the location and assignment decisions are made on an infrastructure network initially constructed without considering future refugee integration. For this reason, modular CCMCP-HC includes capacity decisions to represent a case where the initial infrastructure is designed considering the possibility of integration at the early stages of the crisis. As shown in Table 2, the modular CCMCP-HC delivers the highest schooling rates and capacity utilization for all instances considered.

These results highlight the importance of long-term educational planning in the early responses to a refugee crisis. In the case of Türkiye, educational capacities were allocated as the crisis evolved, and the response efforts have been slow to develop (Cinar and Demir, 2020). Our analyses show that such a myopic approach can decrease capacity utilization and schooling rates. Predicting the number of refugees who will remain in the host country is hard. Even so, our results demonstrate that investments can be made more efficiently as the situation unveiled.

Finally, we would like to discuss the equity aspect of the provided solutions. Educational equity can be defined in two ways: equal outcomes or equal opportunities (Põder et al., 2017). Our proposed approach focuses on enabling schooling access for a greater number of students to provide an equitable educational opportunity to all refugee children. Thus, we introduce an inequity measure that represents each child’s disutility for a given assignment. Schooling distance is commonly used as a metric of inequity in the literature (Felan et al., 2015; Liu et al., 2022). We extend this notion to include the disutility of not being assigned to a schooling option and capture the inequity not only among the assigned students but also among all refugee children in a city. For a child assigned to a schooling option, the disutility is the travel distance to school; for children not assigned, we assign a disutility of k⋅θ , where θ is the maximum allowable bus travel distance between a district and a schooling option. In our computational analysis, we use k=2 , since not having access to education has a greater disutility than any schooling option.

In Table 3, we compare the proposed models with the address-based system regarding the inequity measure. The results demonstrate that CCMCP-HC and modular CCMCP-HC reduce the average and standard deviation of disutility in all instances compared to the existing system, implying that the proposed systems achieve more equitable results. Furthermore, for instances with 9, 10, and 11 central schools, CCMCP-HC and modular CCMCP-HC achieve full coverage, reducing the maximum disutility to ≤100.

**Table 3.** Comparison of the average, minimum, maximum, and standard deviation of disutility obtained by address-based, CCMCP-HC, and modular CCMCP-HC assignments.

		Address-based				CCMCP-HC				Modular CCMCP-HC			
Central Schools	tTECs	Avg.	Min.	Max.	Std.Dev.	Avg.	Min.	Max.	Std. Dev.	Avg.	Min.	Max.	Std. Dev.
3	21	54.06	0	100	46.98	38.43	0	100	45.59	39.26	0	100	43.24
4	21	51.65	0	100	44.58	35.13	0	100	42.25	33.12	0	100	40.86
5	21	45.48	0	100	44.50	29.70	0	100	39.55	28.37	0	100	37.49
6	21	39.63	0	100	43.66	27.09	0	100	34.73	26.08	0	100	32.37
7	21	38.91	0	100	39.82	21.75	0	100	29.34	21.62	0	100	26.78
8	21	33.73	0	100	38.10	18.79	0	100	22.14	13.25	0	100	16.31
9	19	33.52	0	100	36.32	17.76	0	48.72	17.91	20.00	0	44.28	16.96
10	16	33.91	0	100	36.04	19.03	0	48.72	17.54	19.67	0	44.28	16.30
11	14	32.15	0	100	36.50	20.76	0	49.06	17.53	21.62	0	48.76	16.55

Note. CCMCP-HC = cooperative capacitated maximum covering problem with heterogeneity constraints; tTECs = transformed Temporary Education Centers.

### 4.3. Sensitivity Analyses

#### 4.3.1. Impact of Capacity

As we explain in Section 3, the World Bank made ample funds available to increase schooling capacity and set ambitious targets for refugee children’s education. Therefore, we solve all three models with 50% increased central school capacity; in contrast, the TEC capacities stayed the same (see Online Appendix D.1). Two points can be made after comparing the results for increased and existing capacity levels. First, with ample central school capacity, common sense suggests that the benefits of cooperative and modular models would vanish since central schools have the capacity to easily serve more districts. However, our results show that the proposed models perform significantly better than the address-based benchmarking case, regardless of available capacity.

**Figure 2.**
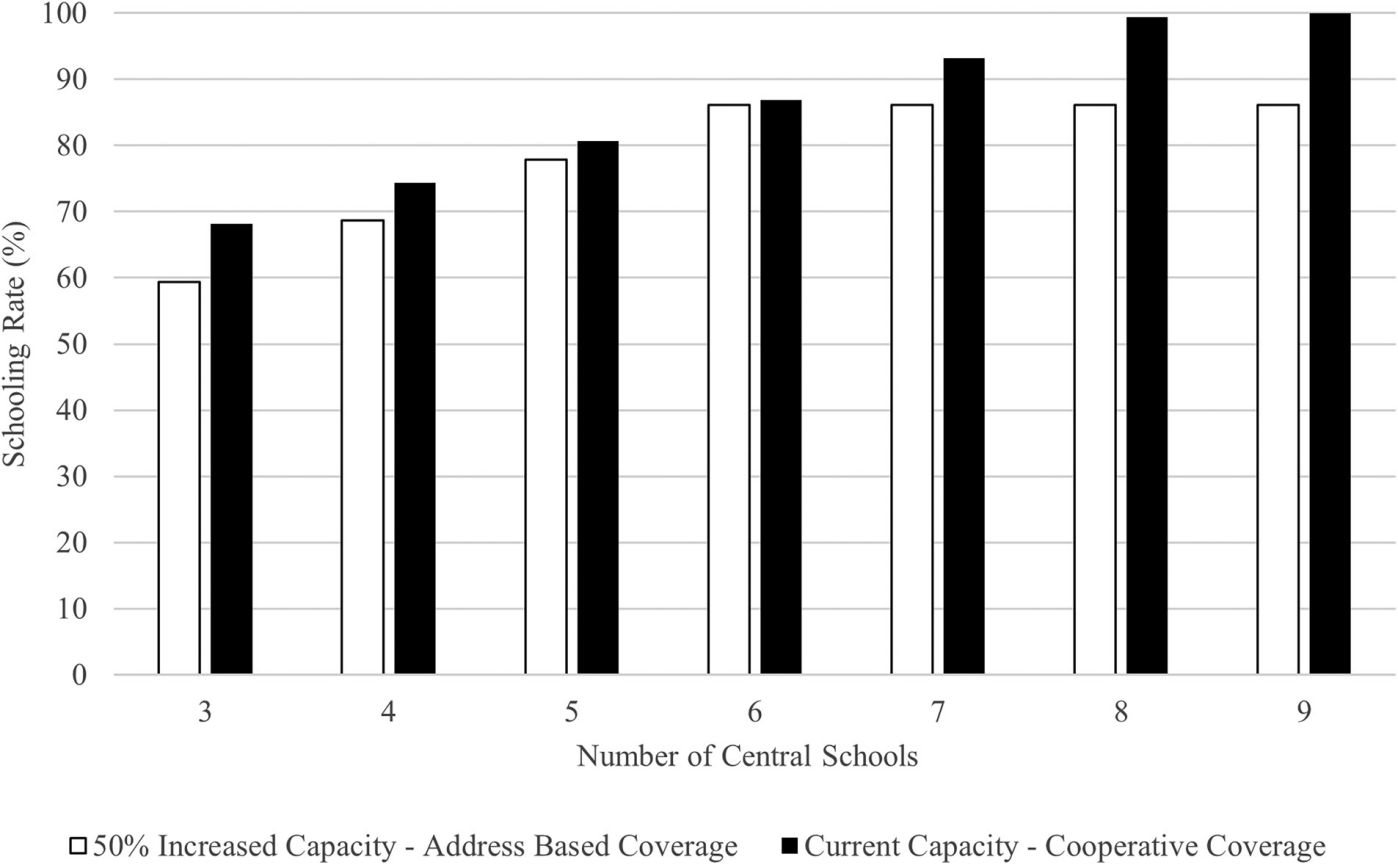
Sensitivity analysis of capacity expansion for different numbers of central schools.

More importantly, our analysis shows that shortcomings of an address-based allocation system cannot be remedied even through considerable capital investment. As seen in Figure 2, even when all the central schools increase their capacity by 50%, the schooling rates plateau in the address-based system (white bars), whereas cooperative coverage (black bars) can achieve higher potential schooling rates utilizing current capacity. The results thus point to a significant policy implication: rather than blindly increasing the capacity of the existing central schools by adding more classrooms, higher schooling coverage can be achieved by (i) introducing a more flexible assignment system and (ii) integrating the existing TECs into the education system through a centralized curriculum and proficient teaching staff. Both interventions are arguably more cost-efficient than building new capacity, and we strongly recommend they be considered at the execution stage of European Union (E.U.) and World Bank-funded projects for refugee schooling.

#### 4.3.2. Impact of the Network Structure

Kilis is the most refugee-dense province of Türkiye; however, it is also very small in area and population. Almost half of the population consists of refugees, and new infrastructure investments have an immediate positive impact on this community. As the inter-district distances are small, schools and TECs can serve a wide area. The composition may be entirely different for other provinces with high refugee populations, especially if refugees remain a minority within the province. In such cases, “urbanization” and the inclusion of refugees may be overlooked. Therefore, it is critical to test the performance of the proposed methodology with different refugee schooling network structures.

We test the robustness of the proposed models through sensitivity analyses over the Gaziantep and Sanliurfa districts, which have the second and fourth highest proportion of refugees among the country’s provinces but are geographically and demographically different from Kilis and each other. Our detailed results are provided in Online Appendix D.2.

The existing infrastructure is clearly insufficient to serve the population in either of the provinces. As a result, their schooling options should be improved. Comparing the results among the three provinces, we can say that the proposed models provide robustly higher coverage, regardless of the network structure. Another key insight is that a priori capacity planning is considerably more important for the regions with scattered refugee populations. The results emphasize the critical role of TECs, especially for semiurban and rural refugee populations.

## 5. Conclusions

Our study reflects on lessons from Türkiye’s provision of education to refugees over 10 years. Access to education is not only a fundamental human right but will also help the next generation of refugees become self-sufficient. Refugees can achieve economic prosperity only through inclusive systems; this is vital for beneficiary graduation, that is, beneficiaries maintaining their lives without financial aid from the host countries and international organizations.

### 5.1. Recommendations and Limitations

In this study, we proposed an alternative approach to reinforce the inclusion of refugee children in the host country’s education system. We designed a strategy to overcome the challenges of integration and improve refugee children’s schooling access without burdening the existing education infrastructure. We demonstrated the benefits of more flexible school assignment policies (cooperative coverage instead of address-based assignments, modular capacity increase, and double shifts). Our results align with existing studies that suggest any proposed mechanism or framework for refugee integration should revisit existing policies and support the right to education for refugee children (Betts and Collier, 2015).

Although this study is based on a decade’s worth of reports and a promising system design, it concerns a complex and evolving humanitarian crisis and, as such, is not without limitations. We conducted our computational analyses with real-life data from three major refugee-hosting provinces in Türkiye and focused on the strategic and tactical problems of capacity allocation and children’s assignments. Operational dynamics can also be optimized (i.e., school bus routing and teacher scheduling) to ensure the proposed policy changes can be realized smoothly; however, such an attempt requires more granular data for each class level. The recommendations of this study could also be tested through longitudinal studies. We proposed an approach that *theoretically* overcomes the barriers to integrating Syrian refugee children into the education system and improves schooling access. However, long-term effects should be investigated in follow-up studies.

Finally, different refugee-hosting countries with distinct compulsory education systems and requirements might require different solutions. For example, in Jordan, double-shift practices were solely introduced for refugees, who were taught by underpaid part-time teachers, yielding a significantly reduced quality of education (Salem, 2019). Without double-shift programs and TECs, countries should identify alternative language training and support channels.

### 5.2. Implications for Ukrainian Refugee Crisis

The lessons from the Syrian case have become invaluable in addressing the Ukrainian crisis. The E.U. called on its members to open up registration in public schools and plan for long-term integration (European Commission, 2022). OECD (2022) acknowledged the challenges faced by the host countries, including the lack of adequate capacity, and urged them to look into the strategies adopted by other Organisation for Economic Co-operation and Development (OECD) countries with refugee experiences, including Türkiye.

Within a few months into the Ukrainian refugee crisis, challenges similar to those in the Syrian case were reported. In Poland, TECs started to provide education to Ukrainian refugees (Ptak, 2022). European Commission (2022) listed lack of interest in learning the host country’s language and war trauma as significant barriers. Very few students (28%) renewed their registrations in Polish courses after Spring 2022, hoping that they would be returning home soon (Reuters, 2022). Ukraine’s refugee children received more generous funding than children in any previous crisis (The Economist, 2022). Regardless, host countries have been quickly overburdened, and education systems are stretched well over their capacity (The New Humanitarian, 2022). Refugee integration will become a critical research topic in the coming years as countries such as Poland, Romania, and Hungary deal with social support systems that are already overwhelmed by the massive influx of Ukrainian refugees. We believe the key considerations outlined here can inform researchers and decision-makers responding to Ukrainian and other refugee crises.

### 5.3. Implications for Operations Management Literature and Future Research Directions

The problem of refugee integration has many facets and can only be addressed through multidisciplinary approaches, including pedagogy, economics, and sociology. However, OR/MS approaches can complement these efforts by developing better logistic networks, resource allocation strategies, and stakeholder coordination. We documented the existing operational challenges associated with refugee schooling during a large-scale refugee crisis and presented a road map for a potential solution. Next, we suggest potential research directions for fostering refugee-inclusive strategies in other contexts.

DEI initiatives have shaped corporate discourse since the turn of the decade, as evidenced by studies exploring the perspectives of DEI-seeking interest groups in the workplace (Joy et al., 2007; Narayanan and Terris, 2020). Nevertheless, little attention has been directed to refugees within management theory and practice (Guo et al., 2020), even if employment is the most crucial factor in their successful integration (Campion, 2018). The United Nations’ call for more active involvement by corporations in achieving the SDGs could be key to addressing issues impacting refugees (United Nations, 2015). It is well documented that climate change (Atasu et al., 2020), supply chain wars, and unsustainable procurement of natural resources (Khanna, 2016) are at the root of refugee crises, such as the food price speculation that sparked the Arab Spring and the Syrian Civil War (Lagi et al., 2011) and large corporate land acquisitions in Africa and Latin America triggering civil unrest (Sassen, 2014). As Kalkanci et al. (2019) argue, further research on sustainable procurement and offshore production practices could help prevent future refugee crises.

Refugees often struggle with securing stable employment and are subject to exclusion, discrimination, sexual harassment, and exploitation (Kroon and Paauwe, 2014); the situation is even more dire for refugee women and girls (Knappert et al., 2018). There is a growing literature on human trafficking (Maass et al., 2020; Dimas et al., 2022; Keskin et al., 2021; Yagci Sokat, 2022; Yagci Sokat and Altay, 2022 and modern slavery in supply chains (Kougkoulos et al., 2021; Cousins et al., 2020); future such studies can investigate the impacts of these practices on members of refugee populations who are marginalized based on their intersecting identities (e.g., gender and disability).

Gericke et al. (2018) find that at the initial integration stage, the support provided by volunteers and social workers is crucial in enabling refugees to navigate their new life in the host country. Unfortunately, early reports show that volunteers responding to the Ukrainian refugee crisis are already on the verge of burnout (The New Humanitarian, 2022). While there are recent studies on volunteer recruitment (Manshadi and Rodilitz, 2022) and management (Urrea et al., 2019) in response to sudden-onset disasters, their potential is yet to be explored in the refugee crises contexts.

## Supplemental Material

sj-pdf-1-pao-10.1177_10591478241243382 - Supplemental material for No Country for Young Refugees: Barriers and Opportunities for Inclusive Refugee Education Practices Supplemental material, sj-pdf-1-pao-10.1177_10591478241243382 for No Country for Young Refugees: Barriers and Opportunities for Inclusive Refugee Education Practices by Sebnem Manolya Demir, Feyza G Sahinyazan, Bahar Y Kara and Elfe Buluc in Production and Operations Management
