# Supplementary material for: No Country for Young Refugees: Barriers and Opportunities for Inclusive Refugee Education Practices
Source: Prod Oper Manag. 2024 Apr 16;34(4):612–26. doi: 10.1177/10591478241243382 (PMC13044447; doi:10.1177/10591478241243382)
Supplement: sj-pdf-1-pao-10.1177_10591478241243382 - Supplemental material for No Country for Young Refugees: Barriers and Opportunities for Inclusive Refugee Education Practices [file sj-pdf-1-pao-10.1177_10591478241243382.pdf]

Addendum

Online Companion - No Country for Young Refugees: Barriers and Opportunities for Inclusive Refugee Education Practices

Sebnem Manolya Demir [1], Feyza G. Sahinyazan [2,*], Bahar Yetis Kara [3], and Elfe Buluc[4]

[1] Marshall School of Business, University of Southern California, Los Angeles, CA, USA, 90089
[2] Beedie School of Business, Simon Fraser University, Vancouver, BC, Canada V6C 1W6
[3] Department of Industrial Engineering, Bilkent University, Ankara, Türkiye, 06800
[4] United Nations High Commissioner for Refugees (UNHCR) Türkiye, Ankara, 06690
* Corresponding Author. feyza_sahinyazan@sfu.ca

Version April 18, 2025

Appendix A. Current school transportation policies of Turkish MoNE for refugee and host country's children

In the study titled "No Country for Young Refugees: Barriers and Opportunities for Inclusive Refugee Education Practices," we discuss certain policy changes to improve access and inclusion of refugee children in educational opportunities. However, such changes may introduce new inequalities among the refugee and host country's children or within these groups. In this subsection, we will explain the existing school transportation regulations for host country's (Türkiye) children.

School buses are commonly used in Türkiye. Accordingly, there are detailed legislations around operational aspects, including which transportation companies are deemed eligible, the main responsibilities of the transport company, the drivers, parents and schools in school transport activities, and qualities sought in school buses, which are determined and overseen by the Turkish MoNE (MoNE 2018b). As cited in our manuscript previously, the coverage radii of the schools are also determined by MoNE (2014) (students cannot travel less than 2 km.s or more than 50 km.s with school buses). This legislation also explicitly mentions that provincial planning commissions ensure smooth access for students affected by disasters, migration, or other accessibility issues (including autism, cerebral palsy, or other disabilities).

On the other hand, not all Turkish students use the school bus option, some can travel by their parents' vehicles, and others choose to use public transport, for which student tariffs are discounted. For other students, who cannot use public transport or need financial support for school buses, there are numerous central (Directorate General of Foundations n.d.), local government (Ankara Metropolitan Municipality 2020) and NGO support programs (Turkish Education Foundation 2023).

School transportation is one of the most cited concerns hampering refugee schooling access by NGO reports and other stakeholders, including teachers, students, and parents. Shamieh et al. (2022) interviewed numerous stakeholders, including Syrian parents, to identify the obstacles hindering their children's education access; they found that one of the main points of concern is the lack of transportation means. Burde et al. (2015) list reducing education costs and the distance to school as critical interventions for improved access.

In their study, Usta et al. (2018) interviewed Syrian teachers in Türkiye. The teachers describe the distance to schooling options as one of the main obstacles that hamper their students' access, as some students need additional funds for school transportation. Coskun and Emin (2016) state, "The difficulty of transportation is another factor concerning access to education. Especially families who are experiencing financial problems cannot afford the resources for transportation and, therefore, cannot send their child or children to school. Classes [in] the afternoon period of public schools end

late, and parents [...] view transportation as a safety issue. As a safety precaution, parents sometimes do not want to send their children to school."

In 2018, when introducing a school bus support program to Turkish MoNE, International Organization for Migration (IOM) (IOM 2018) stated that: "IOM, with funding from the European Union's Civil Protection and Humanitarian Aid Operation (ECHO) and the United States Department of State's Bureau of Population, Refugees and Migration (PRM), addresses a very crucial need – transportation. Syrian children in Türkiye often have a hard time getting to school for a number of reasons: their parents cannot afford to pay for transportation, schools are too far from their homes, or weather conditions make walking difficult."

While numerous programs provide school transportation aid to Syrian children, including IOM's, these reports show other concerns associated with long commute times, such as safety and distance. Furthermore, even if the funding is provided, the parents may not want to entirely rely on these costs, as pointed out by Burde et al. (2015): "It is important to note that the success of such interventions depends in part on how they are structured— whether communities or NGOs or governments bear the costs, and as a result, may not be sustainable in the long run, nor able to serve all populations in need." Indeed, Crul et al. (2019) found that "despite the huge efforts of UN agencies" associated with schooling costs, many families are still struggling.

In summary, school buses are ubiquitous among Turkish and Syrian children. These programs are managed and distributed separately from each other and are funded by different sources. However, it is essential to note that recommendations targeting an underprivileged group should not introduce additional DEI challenges and create further tension between the host nation and the refugees. As an exemplary application, IOM (2019) recently introduced a 25% quota for low-income Turkish students in their school transportation program. We believe this is not an issue in this context, especially since several governmental and non-governmental organizations provide funds for local and refugee student transportation. But, we would like to highlight the importance of accessible transportation options for refugee children, especially given that they are less likely to rely on alternate solutions. Accordingly, in this study, we recommended integrating TECs into the central schooling system as refugee children more conveniently access them. We also demonstrated that more flexible school assignment policies could reduce the disutility caused by lack of access to education and long commute distances.

Appendix B. Details of the Proposed Mathematical Models

Appendix B.1. Cooperative Capacitated Maximal Covering with Heterogenity Constraints (CCMCP-HC)

Our problem is represented over a network of nodes ($\mathcal{N}$), that consists of the districts representing the urban or peri-urban neighbourhoods with high refugee concentration ($\mathcal{D}$) with population $P_i, i \in \mathcal{D}$; candidate central schools: Turkish State Schools with adequate resources for the enrollment of Syrian refugee children in a double-shift formation ($\mathcal{C}$); and the TECs ($\mathcal{D}' \subset \mathcal{D}$). TECs are allocated for the refugee children's education only and therefore located on refugee settlement districts. The school capacities are represented as $Q^T_j, j \in \mathcal{D}'$ and $Q^C_j, j \in \mathcal{C}$ for TECs and central schools, respectively. Distances in the defined network are represented as L_{ij} where $i, j \in \mathcal{N}$.

In addition to heterogeneous facilities, heterogeneous modes of transportation are considered in the proposed formulation. In the classical CMP models, demand points located further than a certain distance threshold to a service facility, i.e., the coverage radius cannot be assigned to that facility. In our case, while a similar assumption holds following the MoNE's regulations, children can either walk to their assigned schools or take the school buses (MoNE 2014). However, the children

who reside within walking distance to their school are not assigned to a state-provided school bus. These two levels of coverage incorporate a multilevel service notion to the formulation instead of an all-or-nothing approach. As we consider heterogeneous facilities, they can also differ in their coverage radii for walking and school bus transportation options. This extension is especially crucial for this particular context as one of the major concerns of the Syrian parents is long commute times, resulting in higher transportation costs. To this extent, imposing a coverage radius as large as the central schools would defy the purpose of transforming TECs, which is providing more accessible schooling options. Consequently, we define shorter allowable walking and bus commute coverage radii for TECs (α, β) than for the central schools, respectively ($\gamma \geq \alpha, \theta \geq \beta$).

Using these coverage radii, we construct sets to represent the TECs and central schools in the allowable walking and bus travel distances to each district $i \in \mathcal{D}$ as ($\mathcal{A}_i, \mathcal{B}_i, \boldsymbol{\Gamma}_i, \boldsymbol{\Theta}_i$). These sets enable the construction of a set-based formulation, which is computationally more efficient than introducing allowable distances as separate constraints for each district-school matching. Furthermore, we define upper bounds on the number of central schools selected (H) and TECs transformed (K). These parameters can be interpreted as budget constraints representing government's budget to support these facilities. These upper bounds can be further lowered to represent the scarcity of teachers with proper formation.

CCMCP-HC is formulated with the objective of maximizing the population served: the total number of refugee children in a district ($i \in \mathcal{D}$) assigned to a schooling option (t_i). CMCP-HC decides on the locations of central schools (z_j) from the set of candidate central schools ($\mathcal{C}$), and the locations of TECs that are integrated into Turkish education system (f_j) from the set of all districts with TECs ($\mathcal{D}'$). We will refer to these integrated TECs as "transformed TECs" (tTECs). As the tTECs are located in refugee-dense districts, we assume that they first serve the children located in that district. Therefore, the number of children from district $j \in \mathcal{D}'$ that can be assigned to a tTEC located in the same district can be represented as $O_j = min\{P_j, Q_j^T\}$. Furthermore, the model determines the number of children utilizing different modes of transportation, whether they are walking to a tTEC (w_{ij}) or to a central school (n_{ij}); or taking the school bus to a tTEC (b_{ij}) or to a central school (s_{ij}). Accordingly, the formulation of CMCP-HC is as follows:

Sets and Parameters:

$\mathcal{N}$: Set of nodes where $\mathcal{N} = \mathcal{D} \cup \mathcal{C}$

$\mathcal{D}$: Set of districts

$\mathcal{D}'$: Set of TECs (candidate tTECs

$\mathcal{C}$: Set of candidate central schools

L_{ij} : Distance between nodes $i \in \mathcal{N}$ and $j \in \mathcal{N}$

P_i : Population of refugee children in district $i \in \mathcal{D}$

Q_j^T : Capacity of TEC located in $j \in \mathcal{D}'$ (number of children)

Q_j^C : Capacity of central school located in $j \in \mathcal{C}$ (number of children)

α : Allowable walking distance between a district and a tTEC

β : Allowable bus travel distance between a district and a tTEC

γ : Allowable walking distance between a district and a central school

θ : Allowable bus travel distance between a district and a central school

$\mathcal{A}_i = \{j \in \mathcal{D}' : L_{ij} \leq \alpha\}$: Set of TECs in the allowable walking distance to district $i \in \mathcal{D}$

$\mathcal{B}_i = \{j \in \mathcal{D}' : L_{ij} \leq \beta\}$: Set of TECs in the allowable bus travel distance to district $i \in \mathcal{D}$

$\boldsymbol{\Gamma}_i = \{j \in \mathcal{C} : L_{ij} \leq \gamma\}$: Set of candidate central schools in the allowable walking distance to district $i \in \mathcal{D}$

$\boldsymbol{\Theta}_i = \{j \in \mathcal{C} : L_{ij} \leq \theta\}$: Set of candidate central schools in the allowable bus travel distance to district $i \in \mathcal{D}$

H : Upper bound on the number of central schools selected

K : Upper bound on the number of tTECs

ψ^C : Lower bound on the number of students that should be assigned to a central school

ψ^T : Lower bound on the number of students that should be assigned to a tTEC

$O_i = min\{P_i, Q_i^T\}$: Number of children from district $i \in \mathcal{D}'$ that can be assigned to a tTEC located in the same district

Decision Variables:

t_i : Number of children in district $i \in \mathcal{D}$ assigned to a schooling option

$$f_i = \begin{cases} 1, & \text{if TEC in the district } i \in \mathcal{D}' \text{ is transformed} \\ 0, & \text{otherwise} \end{cases}$$

$$z_j = \begin{cases} 1, & \text{if candidate school } j \in \mathcal{C} \text{ is selected as a central school} \\ 0, & \text{otherwise} \end{cases}$$

w_{ij} : Number of children walking from district $i \in \mathcal{D}$ to the tTEC in $j \in \mathcal{A}_i$

b_{ij} : Number of children transported by a school bus from district $i \in \mathcal{D}$ to the tTEC in $j \in \mathcal{B}_i$

n_{ij} : Number of children walking from district $i \in \mathcal{D}$ to the central school in $j \in \boldsymbol{\Gamma}_i$

s_{ij} : Number of children transported by a school bus from district $i \in \mathcal{D}$ to the central school in $j \in \boldsymbol{\Theta}_i$

Model 1 - CCMCP-HC:

$$\max \quad \sum_{i \in \mathcal{D}} t_i \tag{1}$$

$$\text{s.t.} \quad O_i \cdot f_i + \sum_{j \in \mathcal{A}_i} w_{ij} + \sum_{j \in \mathcal{B}_i} b_{ij} + \sum_{j \in \boldsymbol{\Gamma}_i} n_{ij} + \sum_{j \in \boldsymbol{\Theta}_i} s_{ij} = t_i \qquad \forall i \in \mathcal{D}' \tag{2}$$

$$\sum_{j \in \mathcal{A}_i} w_{ij} + \sum_{j \in \mathcal{B}_i} b_{ij} + \sum_{j \in \boldsymbol{\Gamma}_i} n_{ij} + \sum_{j \in \boldsymbol{\Theta}_i} s_{ij} = t_i \qquad \forall i \in \mathcal{D} \backslash \mathcal{D}' \tag{3}$$

$$\sum_{j \in \mathcal{C}} z_j \leq H \tag{4}$$

$$\sum_{i \in \mathcal{D}'} f_i \leq K \tag{5}$$

$$\sum_{j \in \mathcal{A}_i} b_{ij} = 0 \qquad \forall i \in \mathcal{D} \tag{6}$$

$$\sum_{j \in \boldsymbol{\Gamma}_i} s_{ij} = 0 \qquad \forall i \in \mathcal{D} \tag{7}$$

$$O_j \cdot f_j + \sum_{i : j \in \mathcal{A}_i} w_{ij} + \sum_{i : j \in \mathcal{B}_i} b_{ij} \leq Q_j^T \cdot f_j \qquad \forall j \in \mathcal{D}' \tag{8}$$

$$\sum_{i:j\in\mathbf{\Gamma}_i} n_{ij} + \sum_{i:j\in\mathbf{\Theta}_i} s_{ij} \leq Q_j^C \cdot z_j \qquad \forall j \in \mathcal{C} \tag{9}$$

$$O_j \cdot f_j + \sum_{i:j\in\mathcal{A}_i} w_{ij} + \sum_{i:j\in\mathcal{B}_i} b_{ij} \geq \psi^T \cdot f_j \qquad \forall j \in \mathcal{D}' \tag{10}$$

$$\sum_{i:j\in\mathbf{\Gamma}_i} n_{ij} + \sum_{i:j\in\mathbf{\Theta}_i} s_{ij} \geq \psi^C \cdot z_j \qquad \forall j \in \mathcal{C} \tag{11}$$

$$t_i \leq P_i \qquad \forall i \in \mathcal{D} \tag{12}$$

$$f_i, z_j \in \{0, 1\} \tag{13}$$

$$t_i, w_{ij}, b_{ij}, n_{ij}, s_{ij} \in \mathbb{Z}^+ \tag{14}$$

The objective function maximizes the number of children assigned to a school. Constraints (2) and (3) determine the assignment of children in districts with and without TECs, respectively. Constraints (4) and (5) are budget constraints for central schools and tTECs, respectively. Constraints (6) and (7) prohibit children from taking the school bus to tTECs and central schools within walking distance, respectively. Constraints (8) and (9) introduce the capacity constraints for tTECs and central schools. Constraint (10) ensures that a TEC is not transformed if a sufficient number of students are not assigned to it. Likewise, Constraint (11) ensures the minimum assignment to central schools. Constraint (12) limits the number of children covered in a district by the population of that district. Constraints (13) and (14) are domain constraints for decision variables.

Appendix B.2. Modular CCMCP-HC

Cooperative CMCP-HC optimizes the integration decisions on an existing infrastructure to address the current situation in Türkiye. However, existing school (candidate schools and TECs) locations and their capacities were constructed gradually in time, in an ad-hoc manner as the refugee crisis evolved. Therefore, resulting infrastructure embodies dysfunctionalities and is far from being optimal. Neither TECs nor state schools were initially constructed with a future plan of integration in mind. We believe that the lessons learned by the Turkish case can and should provide a roadmap for similar situations in the future. With this purpose, we offer a formulation that constructs an infrastructure of central schools and TECs, considering a future integration.

Rather than assuming a fixed and pre-determined capacity, we let the model decide the optimal capacity for each central school q_j^C and tTEC q_j^T in a modular format, by determining the number of classrooms assigned to that facility. Each module or classroom has a capacity of G, and there is a budget of total number of such classrooms to be opened in tTECs and central schools ($\tilde{H}$ and $\tilde{K}$). These modules can also be interpreted as the allocation of teachers with required language skills and pedagogical formation. Note that, $\tilde{O}_j = min\{P_j, G \cdot q_j^T\}$ represents the same dynamics as O_j.

Additional Parameters:

G : Capacity of a single classroom (module)

$\tilde{H}$: Upper bound on the total number of central school classrooms to allocate

$\tilde{K}$: Upper bound on the total number of tTEC classrooms to allocate

Additional Decision Variables:

q_j^C : Number of classrooms assigned to central school at $j \in \mathcal{C}$

q_j^T : Number of classrooms assigned to tTEC at $j \in \mathcal{D}'$

$\tilde{O}_j = min\{P_j, G \cdot q_j^T\}$: Number of children from district $j \in \mathcal{D}'$ that can be assigned to a tTEC located in the same district

Model 2-Modular CCMCP-HC:

$$\max \quad \sum_{i\in\mathcal{D}} t_i \tag{15}$$

$$\text{s.t.} \quad (3), (6), (7), (12), (13), (14)$$

$$\tilde{O}_j + \sum_{j\in\mathcal{A}_i} w_{ij} + \sum_{j\in\mathcal{B}_i} b_{ij} + \sum_{j\in\boldsymbol{\Gamma}_i} n_{ij} + \sum_{j\in\boldsymbol{\Theta}_i} s_{ij} = t_i \qquad \forall i \in \mathcal{D}' \tag{16}$$

$$\sum_{j\in\mathcal{C}} q_j^C \leq \tilde{H} \tag{17}$$

$$\sum_{j\in\mathcal{D}'} q_j^T \leq \tilde{K} \tag{18}$$

$$q_j^C \leq \tilde{H} \cdot z_j \qquad \forall j \in \mathcal{C} \tag{19}$$

$$q_j^T \leq \tilde{K} \cdot f_j \qquad \forall j \in \mathcal{D}' \tag{20}$$

$$\tilde{O}_j + \sum_{i:j\in\mathcal{A}_i} w_{ij} + \sum_{i:j\in\mathcal{B}_i} b_{ij} \leq G \cdot q_j^T \qquad \forall j \in \mathcal{D}' \tag{21}$$

$$\sum_{i:j\in\boldsymbol{\Gamma}_i} n_{ij} + \sum_{i:j\in\boldsymbol{\Theta}_i} s_{ij} \leq G \cdot q_j^C \qquad \forall j \in \mathcal{C} \tag{22}$$

$$\sum_{i:j\in\boldsymbol{\Gamma}_i} n_{ij} + \sum_{i:j\in\boldsymbol{\Theta}_i} s_{ij} \geq z_j \qquad \forall j \in \mathcal{C} \tag{23}$$

$$q_j^T, q_j^C, \tilde{O}_j \in \mathbb{Z}^+ \tag{24}$$

Constraint (16) determines the total number of children assigned to a schooling option. Constraints (17) and (18) represent the budget constraints for classroom assignments. Constraints (19) and (20) allow capacity assignments only to selected central schools and tTECs, respectively. Constraint (21) and (22) are the capacity constrains for tTECs and central schools, respectively. Constraint (23) ensures that a candidate school is not selected as a central school if there are no districts assigned to it. Note that, the value of $\tilde{O}_j$ cannot be determined *a priori* as q_j^T is a decision variable and it introduces a non-linearity to our model. However, this expression can be linearized straight-forwardly.

Appendix C. Description of the Data Sets Used in the Numerical Analyses

Kilis is the province with highest Syrian refugee density, making up approximately 43% of province's total population (Ozdemir 2021). The travelling distances between the districts of Kilis have been adapted from Kian et al. (2020). 43 districts that are densely populated in terms of Syrian refugees are included in the set of districts ($\mathcal{D}$). In our analyses, we focus on high school students, as secondary education level student enrollments demonstrate the lowest schooling rates among all age groups (Dayioglu et al. 2021). In Kilis, there are 15 state high schools that have excess capacity, three of which currently have Syrian students. Their actual locations are represented in the set of candidate central schools ($\mathcal{C}$). There are 21 TECs located in Kilis (MoNE 2018a) and government's plans of closing TECs include all of them. Therefore, we considered all 21 TECs as candidates to be transformed into MoNE operated Turkish State Schools, accordingly, their locations constitute the set for candidate tTECs ($\mathcal{D}'$).

Despite our best efforts to obtain exact numbers of Syrian children at each district, there are no publicly available data for this group due to the high number of unregistered refugees. To circumvent this, for each district, we estimated the number of high-school aged refugee children through the

following procedure: First, we estimated the number of Syrian refugees in Kilis by multiplying Turkish citizen population in the district by Syrian refugees to Turkish citizens ratio (75%) (Ozdemir 2021). Then, we estimated the number of refugee children using the fact that children rate among Syrian refugees located in Türkiye is 47% (UNHCR 2021). Finally, we assumed a uniform distribution among the ages of children (0 to 18 years old) after examining the actual demographic distribution of Turkish and Syrian children (CIA 2022). Accordingly, the proportion of children in high school age corresponds to 22% of all children.

Allowable walking and bus travel distances for central schools (γ, θ) are determined based on MoNE's regulations on Transportation in Education as two and 50 kilometers, respectively (MoNE 2014). Allowable walking distance for tTECs (α) are again assumed to be two kilometers. Allowable bus distance (β) for tTECs is 10 kilometers to sustain their accessibility and locality (Coskun and Emin 2016). For Cooperative CMCP-HC, capacities of central schools (Q_j^C), we used the school capacities reported in Level 3 Statistical Region published by MoNE (2021). With a "double-shift" setting, same number of refugee children can be accommodated. For tTECs capacities, we rely on the fact that approximately 100 thousand children are continuing their education in 224 TECs operating in Türkiye (MoNE 2018a), indicating an average capacity of 450 students per TEC. However, in many cases TECs are serving all the grades at once with reduced schedules. Assuming one third of the school-age children are at the high school age, we estimated a capacity of 150 children per tTEC. We would like also to remind that for the Modular CCMCP-HC, these capacities are decision variables and are determined by the model.

For our sensitivity analyses, we will focus on two other Turkish provinces located at the Syrian border, Gaziantep and Sanliurfa, which have the second and fourth highest refugee proportion in Türkiye, respectively, but are geographically and demographically different from Kilis and each other. Fig. 1 shows these three provinces, their schooling network (districts, TECs and state schools accepting refugee students) and the location of their refugee-dense districts. These districts are identified based on the spatial analyses of refugees conducted over Gaziantep (Sonmez 2016) and Sanliurfa (Karademir and Dogan 2019).

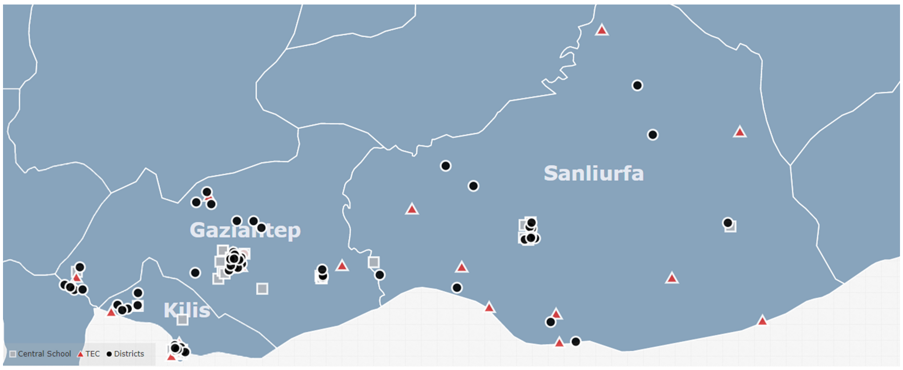

Figure 1. Maps of Kilis, Gaziantep and Sanliurfa for scale

Table 1. Schooling options, demographic statistics, and area comparison of three provinces

	Refugee Schooling		Demography		Area (km^2)
	State Schools	TECs	Refugee Population	Refugee Proportion (%)	
Kilis	15	21	106,000	42.5	1,521
Gaziantep	15	7	461,000	17.8	6,819
Sanliurfa	8	9	428,000	16.7	18,584

Table 1 shows the schooling network, refugee population and their proportion to the province's total population, and area statistics of these three provinces. Based on the network structure and the statistics we can make the following observations. First of all, assuming similar TEC and central school capacities, schooling options available to the refugee children are significantly fewer in Gaziantep and Sanliurfa, in comparison to Kilis, even though the number of refugees in these provinces are comparably higher. One reason is that the refugee camps that are still in operation are located in these provinces. Another reason is that the proportion of the refugees in the total population is much smaller, and as a result, refugee schooling is not considered as a pressing issue.

Second, we observe that the spatial distribution of the refugee dense districts show very different patterns. In Kilis, the refugees are mainly located in the districts that are very close to the border. In Gaziantep, most of the refugees are located near the center of the province, which is reasonable, as Gaziantep ranks as the 10th most vibrant economy among 81 Turkish provinces. In Sanliurfa, the refugee-dense districts are sparsely distributed all over the province, even though there is still some accumulation near the center of the province. Combined with the fact that Sanliurfa has the largest surface area among these three provinces, the schooling options, especially the TECs with their smaller coverage radii, are expected to serve a limited population.

Appendix D. Results of the sensitivity analyses

Appendix D.1. Impact of the capacity

In our previous analyses, we estimated central school capacities as the average of school capacities reported in Level 3 Statistical Region published by MoNE (2021). As refugee children are educated in a "double-shift" program, it is a reasonable assumption that the physical capacity that can be allocated to the refugee children is equal to the existing schooling capacity. On the other hand, given the ambitious target and ample budget provided by the World Bank, the central schools can host far more students in near future. Therefore, we solved all three models with 50% increased capacity for central schools, while the tTEC capacities stay the same, illustrated in Table 2.

With ample central school capacities, one can suggest that the benefits of cooperative and modular models would vanish as the central schools can easily serve more districts within their capacity. However, by contrasting these results obtained by increased and existing capacity levels, our results show that regardless of the capacity levels, the proposed models perform significantly better than the address-based benchmarking case.

Table 2. Sensitivity Analysis for 50% increased central school capacities: Comparison of coverage and utilization rates obtained by CMCP-HC, Cooperative CMCP-HC, and Modular CCMCP-HC

		**CMCP-HC**		**Cooperative CMCP-HC**		**Modular CCMCP-HC**	
**Central Schools**	**tTECs**	**Coverage (%)**	**Utilization (%)**	**Coverage (%)**	**Utilization (%)**	**Coverage (%)**	**Utilization (%)**
3	21	59.39	76.63	74.75	96.44	77.50	100.00
4	21	68.70	79.06	84.14	96.83	86.90	100.00
5	21	77.81	80.81	93.53	97.14	96.29	100.00
6	19	86.11	85.27	100.00	99.02	100.00	99.02
7	15	86.11	85.27	100.00	99.02	100.00	99.02
8	11	86.11	85.27	100.00	99.02	100.00	99.02
9	7	86.11	85.27	100.00	99.02	100.00	99.02

Appendix D.2. Impact of the network structure

Table 3 and Table 4 show the results of sensitivity analyses conducted on Gaziantep and Sanliurfa instances, respectively. In these tables, again, for each central school level, we reported the configuration with the highest coverage. First of all, it is evident that the existing schooling

options are not sufficient to serve the refugee population in neither of the provinces. As a result, the schooling options should be increased for these provinces immediately. Comparing these results with each other and with the Kilis case, we can say that the proposed models (CCMCP-HC and Modular CCMCP-HC) are providing consistently high coverage rates, regardless of the network structure. Another key insight driven by the results is that *a priori* capacity planning has a considerably higher importance for the regions with scattered refugee distribution. For Kilis and Gaziantep, CCMCP-HC and Modular CCMCP-HC provides similar results. However, for Sanliurfa, where refugees are dispersed, the significance of focusing on refugee integration in earlier phases of the crisis is more evident. The highest schooling rate that can be achieved with the existing network is 76.8%, whereas if TEC and central school capacities were initially determined considering a future integration, this rate could have been as high as 91.2%. The transition from the refugee camps to the neighborhoods outside of the camp had been very slow in Sanliurfa in comparison to the other provinces; this time could have been used for proper capacity planning and integration process.

Table 3. Gaziantep Comparison of coverage and utilization rates obtained by address-based, Cooperative CMCP-HC, and Modular CCMCP-HC assignment

		**Address-based**		**Cooperative CMCP-HC**		**Modular CCMCP-HC**	
**Central Schools**	**tTECs**	**Coverage (%)**	**Utilization (%)**	**Coverage (%)**	**Utilization (%)**	**Coverage (%)**	**Utilization (%)**
0	7	5.83	42.38	13.73	99.81	13.76	100.00
1	7	11.07	58.28	18.97	99.86	19.00	100.00
2	7	16.31	67.30	24.21	99.89	24.24	100.00
3	7	21.54	73.07	29.45	99.91	29.48	100.00
4	7	26.77	77.09	34.70	99.92	34.72	100.00
5	7	31.98	80.03	39.94	99.93	39.96	100.00
6	7	37.16	82.20	45.18	99.94	45.20	100.00
7	7	42.28	83.82	50.42	99.95	50.45	100.00
8	7	47.39	85.11	55.66	99.95	55.69	100.00
9	7	52.20	85.68	60.90	99.96	60.93	100.00
10	7	56.60	85.54	66.14	99.96	66.17	100.00
11	7	60.99	85.41	71.38	99.96	71.41	100.00
12	7	64.94	84.72	76.62	99.97	76.65	100.00
13	7	68.66	83.84	81.87	99.97	81.89	100.00
14	7	72.26	82.93	87.11	99.97	87.13	100.00
15	7	75.10	81.30	92.24	99.86	92.37	100.00

Furthermore, coverage rates with no central schools seem to be very low in both Gaziantep and Sanliurfa cases, especially for CMCP-HC. However, a more detailed look into the optimal solution structures for these two cases revealed that the reasons behind these similarly low coverages have different underlying reasons. In Gaziantep, the TECs are located in very high population districts, and their capacities are not well utilized especially with the address-based assignment. Even if we relax the address-based system to increase the utilization, due to the low capacity to population ratio, we do not observe significant increases. To the contrary, in Sanliurfa network, we observe that especially with more flexible capacity allocation policies, the available TECs by themselves can serve a population as high as 39.5%. These results emphasize the critical role that TECs can play, especially for the semi-urban and rural refugee populations.

Table 4. Sanliurfa Comparison of coverage and utilization rates obtained by address-based, Cooperative CMCP-HC, and Modular CCMCP-HC assignments

		**Adress-based**		**Cooperative CMCP-HC**		**Modular CCMCP-HC**	
**Central Schools**	**tTECs**	**Coverage (%)**	**Utilization (%)**	**Coverage (%)**	**Utilization (%)**	**Coverage (%)**	**Utilization (%)**
0	9	3.54	8.98	25.46	64.53	39.45	100.00
1	9	10.54	22.68	32.47	69.89	46.47	100.00
2	9	17.46	32.66	39.49	73.84	53.48	100.00
3	9	24.29	40.14	46.50	76.87	60.49	100.00
4	9	28.34	41.97	53.52	79.27	67.51	100.00
5	9	31.54	42.33	60.53	81.22	74.52	100.00
6	9	33.95	41.63	67.54	82.84	81.54	100.00
7	9	36.21	40.89	74.56	84.20	88.55	100.00
8	9	36.21	37.89	76.82	84.25	91.18	100.00

References

Ankara Metropolitan Municipality. Feb. 2020. Municipality School Bus Support. URL: https://www.ankara.bel.tr/haberler/buyuksehirden-okul-servisi-destegi-13003.

Burde, D. et al. Oct. 2015. What works to promote children's educational access, quality of learning, and wellbeing in crisis-affected contexts. Tech. rep. United Kingdom Department for International Development. URL: https://assets.publishing.service.gov.uk/media/57a0897ee5274a31e00000e0/61127-Education-in-Emergencies-Rigorous-Review_FINAL_2015_10_26.pdf.

CIA. 2022. The World Factbook. URL: https://www.cia.gov/the-world-factbook/about/archives/.

Coskun, I. and M. N. Emin. 2016. A road map for the education of Syrians. Tech. rep., 52. URL: http://file.setav.org/Files/Pdf/20160909223717_a-road-map-for-the-education-of-syrians-in-turkey-pdf.pdf.

Crul, M. et al. Dec. 2019. How the different policies and school systems affect the inclusion of Syrian refugee children in Sweden, Germany, Greece, Lebanon and Turkey. *Comparative Migration Studies* 7 (1), 1–20.

Dayioglu, M., M. G. Kirdar, and I. Koc. 2021. The making of a lost generation: Child labor among Syrian Refugees in Turkey. *SSRN*.

Directorate General of Foundations. N.d. Schooling Support and Scholarship Services, In Turkish: Eğitim Yardımı ve Burs Hizmetleri. URL: https://www.vgm.gov.tr/faaliyetler/hayir-hizmetleri/egitim-yardimi-ve-burs-hizmetleri.

IOM. 2018. Bus Rides Boost Enrolment of Syrian Children in Turkish Schools | IOM Türkiye. URL: https://turkiye.iom.int/stories/bus-rides-boost-enrolment-syrian-children-turkish-schools.

— 2019. IOM Turkey Refugee Response Programme. Tech. rep. UN International Organization for Migration. URL: https://turkiye.iom.int/sites/g/files/tmzbdl1061/files/documents/School_Transportation_One_Pager_Web.pdf.

Karademir, D. and M. Dogan. 2019. Spatial Analysis of Syrian Refugees: The Sanliurfa Case. *Journal of Geography* 39, 111–124.

Kian, R. et al. 2020. Dataset: Syrian refugees in Kilis (Southern Turkey): locations, distances and populations, Mendeley Data, V1.

MoNE. 2014. Ministry of National Education: Regulation on access to education by transportation (In Turkish- Milli Egitim Bakanligi Tasima Yoluyla Egitime Erisim Yonetmeligi). Tech. rep. Ankara: Turkish Ministry of National Education. URL: http://www.meb.gov.tr/meb_iys_dosyalar/2014_09/12024357_tasimayonetmeligi.pdf.

— 2018a. Education services for students under temporary protection (In Turkish - Gecici koruma kapsami altindaki ogrencilerin egitim hizmetleri). Tech. rep. Ankara, 6. URL: https://hbogm.meb.gov.tr/meb_iys_dosyalar/2018_10/23093037_22-Ekim_-2018__Ynternet_BYlteni.pdf.

— 2018b. Procedures and Principles for Operating School Service Vehicles, In Turkish: Okul Servis Araclarinin Calistirilmasina Iliskin Usul Ve Esaslar. URL: http://mevzuat.meb.gov.tr/dosyalar/1959.pdf.

— Sept. 2021. National Education Statistics Formal Education 2020-2021. Tech. rep. Ankara: MoNE, 32. URL: https://sgb.meb.gov.tr/meb_iys_dosyalar/2021_09/10141326_meb_istatistikleri_orgun_egitim_2020_2021.pdf.

Ozdemir, A. 2021. Number of Syrian Refugees in Turkey (In Turkish -Turkiye'deki Multeci Sayisi). URL: https://multeciler.org.tr/turkiyedeki-suriyeli-sayisi/#:~:text=T%C3%BCrkiye'de%20kay%C4%B1t%20alt%C4%B1na%20al%C4%B1nm%C4%B1%C5%9F,736%20bin%2091%20ki%C5%9Fi%20oldu..

Shamieh, J. M., I. H. Sawalha, and H. M. Madanat. Jan. 2022. Vulnerability and Capacity Assessment for refugee education crises. *International Journal of Emerging Markets* 17 (1), 253–276.

Sonmez, M. E. 2016. Spatial distribution and futurity of Syrian refugees in the city of Gaziantep. In: *TÜCAUM International Geography Symposium*. October. Ankara, 392–400.

Turkish Education Foundation. 2023. TEV - Scholarships - Domestic Scholarships. URL: https://www.tev.org.tr/scholarship/en/4/Domestic-Scholarships.

UNHCR. June 2021. 10 Years On, Turkey Continues Its Support for an Ever-Growing Number of Syrian Refugees. URL: https://www.worldbank.org/en/news/feature/2021/06/22/10-years-on-turkey-continues-its-support-for-an-ever-growing-number-of-syrian-refugees.

Usta, M. E. et al. 2018. An analysis of the challenges faced by Syrian teachers in Temporary Education Centers (In Turkish - Suriyeli Egitimcilerin Gecici Egitim Merkezlerinde Karsilastiklari Sorunlarin Incelenmesi). *Mukaddime* 9 (1), 173–188.
